# The Relationship Between Lower Limb Bone and Muscle in Military Recruits, Response to Physical Training, and Influence of Smoking Status

**DOI:** 10.1038/srep09323

**Published:** 2015-03-20

**Authors:** Zudin Puthucheary, Mehdi Kordi, Jai Rawal, Kyriacos I. Eleftheriou, John Payne, Hugh E. Montgomery

**Affiliations:** 1 UCL Institute for Human Health and Performance, and NIHR University College London Hospitals Biomedical Research Centre, London, UK; 2 Division of Respiratory and Critical Care Medicine, University Medicine Cluster, National University Health System, Singapore; Department of Medicine, Yong Loo Lin School of Medicine, National University of Singapore, Singapore; 3 English Institute of Sport, Manchester; and Department of Sport, Exercise and Rehabilitation, Faculty of Health and Life Sciences,Northumbria University, Newcastle upon Tyne, UK; 4 Hippocrateon Private Hospital, Psaron 6-12, Engomi 2408, Nicosia, Cyprus; 5 Scottish National Advanced Heart Failure Service, Golden Jubilee National Hospital, Cydebank, Glasgow, UK

## Abstract

The relationship between bone and skeletal muscle mass may be affected by physical training. No studies have prospectively examined the bone and skeletal muscle responses to a short controlled exercise-training programme. We hypothesised that a short exercise-training period would affect muscle and bone mass together. Methods: Femoral bone and *Rectus femoris* Volumes (RF_VOL_) were determined by magnetic resonance imaging in 215 healthy army recruits, and bone mineral density (BMD) by Dual X-Ray Absorptiometry (DXA) and repeated after 12 weeks of regulated physical training. Results: Pre-training, RF_VOL_ was smaller in smokers than non-smokers (100.9 ± 20.2 vs. 108.7 ± 24.5, p = 0.018; 96.2 ± 16.9 vs. 104.8 ± 21.3, p = 0.002 for dominant/non-dominant limbs), although increases in RF_VOL_ with training (of 14.2 ± 14.5% and 13.2 ± 15.6%] respectively, p < 0.001) were independent of prior smoking status. Pre-training RF_VOL_ was related to bone cortical volume (r^2^ = 0.21 and 0.30, p < 0.001 for dominant and non-dominant legs), and specifically to periosteal (r^2^ = 0.21 and 0.23, p < 0.001) volume. Pre-training dominant RF_VOL_ was independently associated with Total Hip BMD (p < 0.001). Training-related increases in RF_VOL_ and bone volumes were related. Whilst smokers demonstrated lower muscle mass than non-smokers, differences were abolished with training. Training-related increases in muscle mass were related to increases in periosteal bone volume in both dominant and non-dominant legs.

Human regional muscle mass and strength have been shown to be related to local bone mineral density (BMD) and mass in several *cross-sectional* studies. Proximally, the dry weight of the human fourth lumbar vertebra correlates strongly with psoas muscle mass^1^ and back extensor muscle strength correlates with lumbar BMD in postmenopausal women^2^. In limbs, hamstring torque is associated with femoral BMD in younger women^3^, and upper limb lean mass with upper arm bone mass in hemiparetic patients^4^. Local muscle mass and BMD may be better correlated than those at more distant sites^5^, though distant correlation do exist^6^. The ratio of total muscle weight to total bone weight varies little at autopsy^7^,^8^. The relationship between local muscle strength and BMD is, however, weaker in more intensively active young men^9^.

*Prospective* human data also support a relationship between bone mineralization and mass, with skeletal muscle mass. In response to short-term (8 month) loading, both muscle mass and bone mineral content increase in young males^10^. In the longer term, both muscle and bone mass increase in patients with rheumatoid arthritis over a 2-year strength training programme^11^, whilst the increase in lean muscle mass was the best predictor of gain in femoral bone content and density in exercising prepubertal males over 3 years^12^.

The relationship between muscle and bone mass may be explained in a number of ways.

Firstly, Wolff's Law suggests that bones constantly remodel in response to alterations in their environmental mechanical load^13^,^14^,^15^. Thus, skeletal muscle contraction during exercise will cause it to grow, whilst applying forces trophic to bone. These latter effects may supplement other tropic effects on bone which result from direct loading (e.g. ground force). Such effects might account for regional and sport-specific differences in bone mineralisation^9^.

Secondly, the ‘Mechanostat Theory' proposes that the skeleton adapts to the increasing mechanical loads imposed by muscle growing in response to exercise^16^,^17^,^18^,^19^. In support, pubertal gains in BMD are related to those in lean body mass and their velocity of attainment, and generally precede them^20^. However, studies of the dominant and non-dominant arms of tennis players suggest that other factors are involved^21^.

Finally, variation in genes which influence *both* bone *and* muscle responses may partly account for their similar responses to exertional load^20^,^22^ especially given that muscle cells and osteoblasts share a common mesenchymal precursor. Both human^23^,^24^,^25^ and murine^24^,^25^ studies are supportive of such a shared genetic influence^22^,^26^. Whilst major loci with such influence have yet to be identified, efforts to identify them have been strongly endorsed^24^.

Environmental factors such as tobacco smoking have been associated with bone and muscle loss in older populations^27^,^28^,^29^,^30^. However, no studies have explored the impact of short-term physical training on the relationship between skeletal muscle and bone mass. Nor has the impact of training on this relationship been explored using high resolution measures of muscle growth and bone morphology in one anatomical region. The influence of environmental factors (alcohol and smoking) on this relationship likewise remains unexplored. We thus sought to perform such a study, examining the relationship between *rectus femoris* muscle growth and changes in femoral BMD and geometry in young male military recruits exposed to an identical programme of physical training. Given that both smoking and alcohol intake may affect skeletal muscle and bone mass, we also explored their influence of smoking habit and alcohol consumption on these measures.

We have previously reported the impact of environmental and lifestyle factors, and the impact of physical training, on the femoral bone phenotypes of consecutive Caucasian males recruited to the British Army Training Regiment^31^. In part, this involved magnetic resonance imaging of the upper femur. We have now performed a new analysis of acquired images to quantify skeletal muscle volumes in this region, reporting for the first time data relating to skeletal muscle growth in these subjects. We have thence performed novel analysis of the relationship between such growth and remodeling of the femur in the same region.

## Methods

The study had appropriate ethics approval from the Defence Medical Services Clinical Research Committee (DMSCRC), and was carried out in accordance with DMSCRC guidelines and regulation on human research, and met the ethical standards of the 1964 Declaration of Helsinki. Written informed consent was obtained from all subjects. The study structure has been previously described in detail^27^,^32^, but key elements are reiterated or summarised below.

### Subjects

Subjects were drawn from consecutive Caucasian male recruits to the Army Training Regiment, Lichfield, United Kingdom over a 21-month period. Intakes vary in size and timing, but on average some 20 or more individuals enter training every two weeks. Training structure did not change over this timescale. All were free of medication and of significant self-reported or clinically evident musculoskeletal, cardiovascular or renal disease. Height, weight and leg dominance (ball-kicking) were documented at entry, and prior to any formal exercise training being undertaken. Lifestyle factors were documented by self-assessment questionnaire: *Smoking status*(non-, current-, long-term ex- [quitting >6 months prior to enrolment], and recent ex-smoker [quit ≤6 months prior to enrolment]) and habitual *alcohol consumption*(no, low [1–9], moderate [12–21] or high [>21] units wk^−1^ intake) were documented, So, too, was regular *physical activity in the past 5 years*: weight-bearing sports undertaken for ≥1 year and their estimated weekly hours of participation were used to derive an ‘index of activity' (number of sports × weekly hours of engagement). Subjects were classified as light, moderate or heavy exercisers (score ≤19, 20 to 99, and >100 respectively)^27^.

All then underwent an identical intensive twelve-week period of physical training which has been previously described in detail^31^,^33^,^34^. In brief, this involved 28 × 40–80 min periods of strength training (including leg press and dead lift), 15 endurance training episodes (including interval running, and incrementally-loaded marching), and a total of 24 periods of agility training, material handling, circuit training (high-repetition, low-force exercise of all major muscle groups), and sports periods of ball games in a small area. In addition, training included other physical exercise, such as prolonged marching with various loads while on military exercise, and many 40- to 80-min periods of drill that averaged about one 40-min period/day.

### Lower Limb Imaging

Given that UK army recruit training emphasises lower limb strength and endurance training (above), the lower limb was studied. The upper thigh was imaged, as femoral macroscopic architecture and related muscle mass is more readily defined than is the case in the lower femur. All imaging was performed using the same equipment for all subjects.

*Magnetic Resonance Imaging (MRI)* of the thighs was performed at entry and again at the end of training, using a mobile 1.5-T Siemens Sonata MR scanner (Sonata, Siemens Medical Systems, Erlangen, Germany). Subjects were supine, with legs strapped to prevent movement. Subsequently, ten transaxial spin echo images of both thighs (TR669, field of view 45 cm × 45 cm, slice thickness 10 mm) were obtained at 10-mm intervals, with *slice 1* being proximal and just below the level of the lesser trochanter. Images were optimised for assessment of bone volumes, whilst also capturing images of surrounding skeletal muscle.

As previously described, femoral bone volumes were assessed by one clinician, using CMRtools (Cardiovascular Imaging Solutions, London, UK), for the upper five slices where (for technical reasons) image quality was best. In this way, femoral, endosteal (medullary), periosteal and cortical (endosteal + periosteal) volumes for the 50 mm section of each femur (PV, EV and CV respectively) were calculated. Measurements for the dominant and non-dominant sides were averaged to provide individual mean PV, EV and CV values.

Meanwhile, surface area and volume measurements were made of *rectus femoris* muscle (part of the quadriceps femoris muscle group in the anterior compartment of the thigh) for each of the ten slices in each leg (20 per subject). Image analysis was performed using OsiriX Imaging Software™ version 5.7.1 (open source, Geneva, Switzerland). *Rectus femoris* was defined by the boundary between (black) epimysium and (homogenous grey) skeletal muscle. For the purpose of analysis, the images were magnified 300-fold onscreen. Muscle volume for each slice was calculated from the measured muscle cross-sectional area; given that slice thickness was 10 mm. The sum of image volumes was then recorded as total measured muscle volume for the limb. Inter-slice volume was not estimated. Where image quality did not have sufficient contrast or resolution to enable accurate muscle delineation in all slices, that subject was excluded from analysis. Inter-observer error of the drawing technique was investigated in 7 separate operators. Each drew around the *rectus femoris* muscle, repeating the process on 5 occasions for each of ten slices. The interclass correlation coefficient was 0.995 (0.9931 to 0.9966 95% confidence interval). A single operator (MK, with intra-observer correlation coefficient 0.9962 [0.992 to 0.998 95% confidence interval]) then analysed all muscle volumes.

### Bone Mineral Density

Hip Bone Mineral Density (BMD) was assessed (as previously described^27^,^31^ by dual x-ray absorptiometry (Hologic QDR-1000/W system; Hologic Inc,Bedford, MA, USA) using analysis protocols and edge detection algorithms (VERTEC Scientific Ltd, Reading, UK). Subjects were supine, with the foot braced and strapped to a plastic triangular frame, ensuring fixed internal rotation of 60°. Regional and net average BMD measurements for of the left total hip (THBMD), femoral neck(FNBMD), trochanter (TRBMD), inter-trochanteric region(ITBMD) and Ward's area (WTBMD). The BMD of an 8-cm segment of the proximal femur immediately distal to the base of the lesser trochanter was also obtained (PFBMD). A quality control programme that includes use of ananthropomorphic phantom was run at the start of each scan session.

### Statistical Methods

All data were assessed for normality using D'Agostino and Pearson omnibus normality tests. Parametric data were then analysed using Student's t-test, and non-parametric data were analysed with Pearson's coefficient, Mann-Whitney U test and Wilcoxon's signed Rank Tests as appropriate. Univariate linear and logistic regression analyses were applied (Statistical Package for the Social Sciences version 17 (SPSS, Inc, Chicago, Ill)). Backward multivariate linear and logistic regressions were performed with univariate linear and logistic screening- all variables with p < 0.10 were entered into the multivariate analyses. Where data were non-normally distributed data were log-transformed and the results assessed for normality prior to entry into regression analyses. Between group differences for alcohol intake, smoking status and weight bearing activity was calculated using one-way analysis of variance (ANOVA). Parametric variables were reported as mean (standard deviation); and nonparametric variables, as medians (ranges). Statistical significance was reported for *p* < 0.05.

## Results

**Figure 1:**
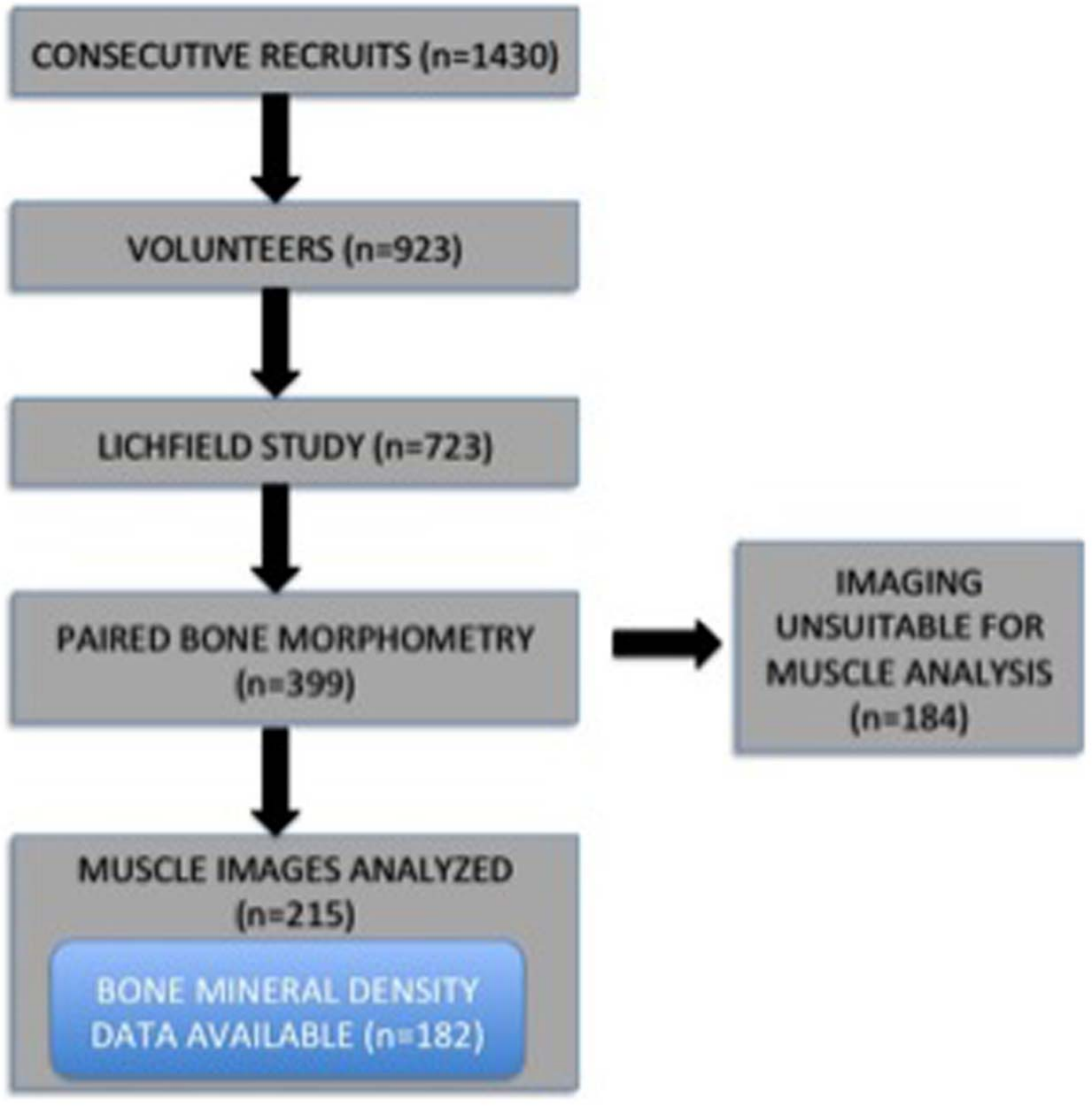
Flowchart of paired muscle and bone image analysis subcohort.

Seven hundred and twenty-three subjects entered the study, 399 of whom had paired MRI images suitable for assessment of bone morphometry^27^. Of these, image quality was suitable for muscle analysis in 215, whose baseline anthropomorphic measurements (age 20.0 ± 2.3 years, height 178.1 ± 5.9 centimetres (cm), weight 73.8 ± 9.7 kilograms (kg), body mass index (BMI) 23.2 ± 2.6) did not differ from the 184 whose muscles were not analysed (p > 0.20 in all cases) (table 1). One hundred and eighty two of these also had BMD data available (Figure 1).

### Bone Phenotypes

Bone volume data at entry (Table 2) were representative of the larger sample set from which they were drawn (p > 0.2 for all measures)^31^), and were not related to age, weight or BMI(p > 0.2). Correlations were seen between height and all bone volumes (periosteal volume r^2^ = 0.27, endosteal volume r^2^ = 0.17 and cortical volume r^2^ = 0.10, all p < 0.001). In the larger cohort (n = 723), we had previously shown past exercise burden to be associated with greater cortical and periosteal bone volume^27^. In this smaller subset (likely due to subsequently reduced power), no relationship was seen with smoking, exercise or alcohol history (p > 0.2).

Both PV and CV increased with training (*P* < 0.001) and to a similar degree in both legs, whilst EV did not alter (*P* = 0.66, Table 2). The change in CV and PV were related to subject height (r^2^ = 0.02, p = 0.03 in each case).

Femoral BMD data were also consistent with the larger sample set from which they were drawn^31^ (p > 0.2 for all measures), and rose with training in all areas assessed (Table 2).

### Muscle Volumes

Prior to training, *rectus femoris* volume (RF_VOL_) was greater for the dominant than non-dominant legs (104.5 ± 22.3 mm^3^ vs. 100.3 ± 19.5 mm^3^, p = 0.02). RF_VOL_ in both legs were associated with height (dominant r^2^ = 0.1, p < 0.001, non-dominant r^2^ = 0.1, p < 0.001) and weight (dominant r^2^ = 0.241, p < 0.001; non-dominant r^2^ = 0.243, p < 0.001), and thus with BMI (dominant r^2^ = 0.16, p < 0.001, non-dominant r^2^ = 0.16, p < 0.001). In both dominant and non-dominant limbs, RF_VOL_ was unrelated to category of alcohol intake or (in the 144 for whom relevant data were available) to history of past exercise. However, RF_VOL_ was smaller in those with a positive smoking history when compared to non-smokers in both the dominant (100.9 ± 20.2 vs. 108.7 ± 24.5, p = 0.018) and non-dominant (96.2 ± 16.9 vs.104.8 ± 21.3, p = 0.002) limbs. This association was still present once corrected for BMI (dominant p = 0.035, non-dominant p = 0.006).

**Figure 2:**
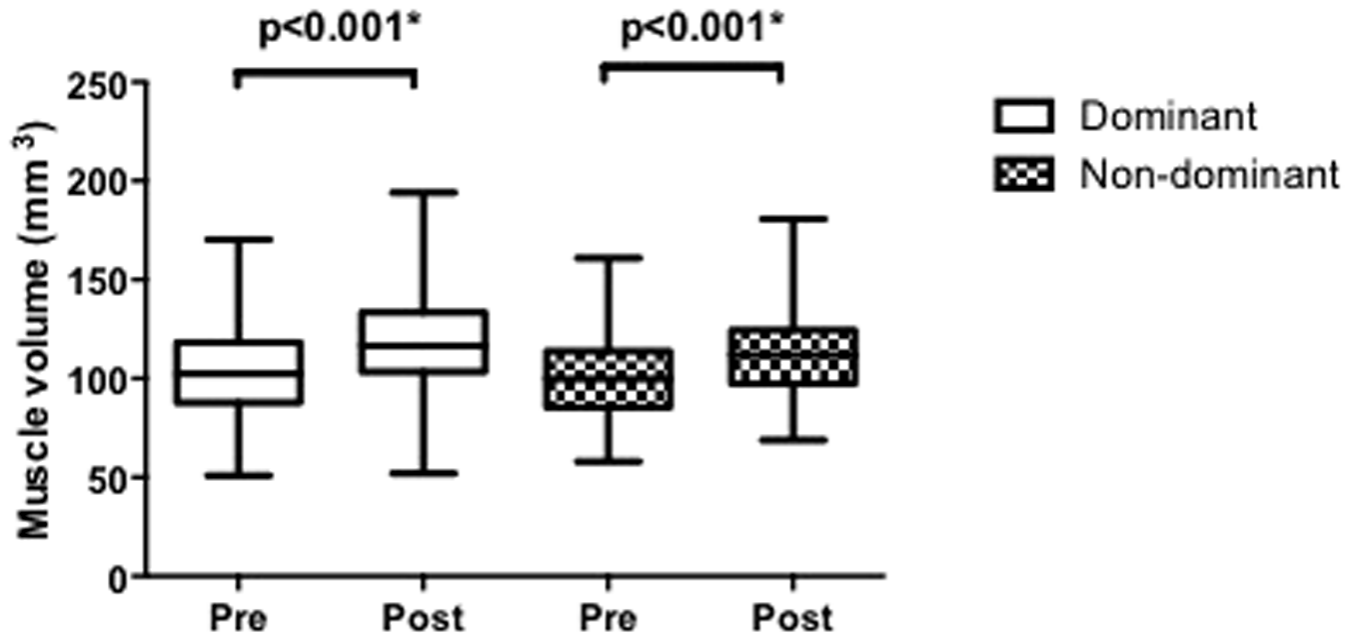
Change in *rectus femoris* muscle volume with military training, in dominant and non-dominant limbs. * denotes p < 0.05. Box and Whisker plots are of median and range.

In response to training, RF_VOL_ increased in both dominant (104.5 ± 22.3 to 117.6 ± 21.5, p < 0.001) and non-dominant (100.3 ± 19.6 to 112.5 ± 21.3, p < 0.001) limbs (figure 2), a rise of 14.2 ± 14.5% and 13.2 ± 15.6% respectively. Change in RF_VOL_ (ΔRF_VOL_) between limbs was highly correlated (r^2^ = 0.78, p < 0.001).

Percentage change in RF_VOL_(%ΔRF_VOL_) in both dominant and non-dominant limbs was associated with BMI (r^2^ = 0.032, p = 0.008 and r^2^ = 0.026, p = 0.017) and initial RF_VOL_ (r^2^ = 0.128, p < 0.001 and r^2^ = 0.159, p < 0.001). In a multivariate linear regression, only initial RF_VOL_ was retained. In a logistical regression, smoking history was not associated with %ΔRF_VOL_ (p > 0.05 for both limbs). Further, no significant differences were seen in post-training RF_VOL_ between smokers and non-smokers in either the dominant (115.2 ± 19.6 vs.119.5 ± 24.1, p = 0.18) or non-dominant (109.1 ± 19.2 vs. 114.8 ± 23.6, p = 0.20) limbs, even when corrected for BMI (p > 0.05 for both limbs). Neither previous alcohol intake nor exercise history were associated with %ΔRF_VOL_ (p > 0.05 for both limbs).

### Bone-Muscle Relationships

#### Baseline values

At baseline, dominant leg RF_VOL_ was related to bone cortical volume (r^2^ = 0.21, p < 0.001), likely resulting from an association with periosteal volumes (r^2^ = 0.21, p < 0.001) rather than endosteal volume (r^2^ = 0.01, p = 0.22). The same held true in the non-dominant leg (r^2^ = 0.30, p < 0.001; r^2^ = 0.23, p < 0.001; r^2^ = 0.00, p = 0.41 for relationship with cortical, periosteal and endosteal volumes respectively).

RF_VOL_ was associated with all measures of femoral BMD, of all 4 parts of the femur measured, but only with Total Hip BMD after multivariate linear regression (p < 0.001, Table 3).

Baseline cortical bone volume was associated with RF_VOL_, in both dominant (r^2^ = 0.214, p < 0.001) and non-dominant legs (r^2^ = 0.296, p < 0.001), this being predominantly the result of periosteal (p < 0.001) rather than endosteal volume (p > 0.20).

The association between RF_VOL_ and Total Hip BMD was independent of smoking history. Associations were seen within all levels of alcohol intake (all p < 0.05) except for abstinence (p > 0.2). Total Hip BMD was associated with RF_VOL_ in those with low (r^2^ = 0.19, p < 0.001) and medium activity (r^2^ = 0.27, p < 0.001) levels but not high (r^2^ = 0.35, p = 0.054). When corrected for BMI, RF_VOL_ remained associated with cortical bone volume (n = 173,r^2^ = 0.09, p < 0.001) and with Total Hip BMD (n = 183, r^2^ = 0.17, p < 0.001).

#### Response to training

Except for cortical volume in the non-dominant leg, change in RF_VOL_ was related to increases in all bone volumes in both legs (Table 4). A weak association was seen between %ΔRF_VOL_ and total hip BMD (r^2^ = 0.049, p = 0.003) and with proximal femur BMD (r^2^ = 0.048, p = 0.003) but became non-significant when corrected for BMI (total hip BMD r^2^ = 0.00, p = 0.53; proximal femur BMD r^2^ = 0.00, p = 0.64).

When corrected for weight bearing activity variable relationships between %ΔRF_VOL_ and %Δbone volumes were seen. A significant relationship was seen in the dominant leg in low (n = 64, r^2^ = 0.20, p < 0.001) and medium (n = 62, r^2^ = 0.39, p < 0.001) activity groups but not in high activity (n = 11, r^2^ = 0.28, p = 0.09), in which a relationship was seen with the non-dominant leg only (r^2^ = 0.56, p = 0.01).

## Discussion

In this prospective study of regional bone and muscle mass, muscle volume was related to bone mineral density and volume, both at baseline and in response to training.

Prior to training, *rectus femoris* volume was greater for the dominant than non-dominant limb and positively associated with height and weight (and thus BMI). Training-related increases in RF_VOL_ in the dominant and non-dominant limbs were highly correlated, and only initial RF_VOL_ appeared an independent determinant of muscle response to training.

Height correlated with all bone volumes at entry and with their training-related change, albeit that the proportion of variation attributable to height was low (r^2^ variably 0.02–0.27). Similarly, height and weight (and thus BMI) also correlated with initial muscle volumes, but again accounted for a limited proportion of variation in these phenotypes (r^2^ = 0.10, 0.24 and 0.16 respectively). Impacts on change in muscle volume were smaller still, given r^2^ values of 0.26–0.32, although initial muscle volume perhaps exerted a greater influence on such change (r^2^ = 0.13–16).

In terms of the relationships between muscle and bone volumes, similar associations (again accounting for small proportions in phenotypic variation) were observed (r^2^ = 0.01–0.30). Likewise, contributions of muscle volume to variance in BMD were small (given r^2^ = 0.17) and smaller still when the association of %ΔRF_VOL_ with BMD was addressed (r^2^ = 0.049).

The relationship between changes in muscle and bone volumes was stronger for moderate than low habitual activity (r^2^ = 0.20, and 0.3 respectively) - and very strong in the dominant leg of those of high activity status (r^2^ = 0.56).

Whilst the observations that muscle volume is related to BMI and is greater in the dominant limb, and that the growth responses in both limbs are correlated, are perhaps unsurprising, other observations are of greater interest. RF_VOL_ was smaller in smokers than non-smokers even after adjustment for BMI. Ours is the first study to confirm a relationship between smoking history and reduced muscle mass in young healthy individuals. Data from an Italian study did perhaps suggest this association, but failed to reach statistical significance^35^. Nonetheless, the observation is in keeping with the association of smoking-related chronic lung disease (chronic obstructive pulmonary disease, COPD) with reduced muscle mass^36^,^37^ and with muscle damage independent of disease severity and treatment^38^. It is also consistent with reduced measures of muscle strength, and with the increased prevalence of sarcopaenia amongst elderly smokers^39^. Further, the association is biologically plausible: fractional protein synthesis rates are lower in the skeletal muscle of smokers, and are associated with increased expression of (growth-inhibiting) Myostatin and members of the protein catabolic pathway as Atrogin-1^40^. Whether the lack of relationship between RF_VOL_ response to training and smoking history reflects a lack of biological effect, the magnitude of training response exceeding that of smoking (making our study underpowered to detect an effect), or the consequence of smoking habit changing substantially during the training period, cannot be determined.

Prior to training, RF_VOL_ was related to both bone mineral density and bone geometry. It was related to bone cortical volume in both the dominant and non-dominant legs, and specifically to periosteal rather than endosteal volume. Such bone geometric change is likely to increase bone strength, meaning that both bone and muscle strength are related. Such findings support a relationship between bone geometry and muscle mass^30^,^41^. A similar relationship with Total Hip BMD remained after multivariate linear regression. Such data are consistent with those reported by others: muscle wasting and loss of bone cortical thickness follow motor paresis in rats and bone weight in kittens^42^. Human regional muscle mass and bone mass^1^ and BMD^2^,^3^,^4^ are related, whilst global muscle mass correlates with BMD at diverse sites and the ratio of total muscle weight to total bone weight varies little^6^,^7^,^8^.

The bone and muscle responses to training were also related. Except for cortical volume in the non-dominant leg, training-related change in RF_VOL_ was related to increases in all bone volumes in both legs. No associations were seen between change in RF_VOL_ and change in bone mineral density.

These data support the co-regulation of human bone and muscle mass and architecture. They do not offer insight into whether these result from a response to shared loading stimuli, from the influence of skeletal muscle contractile force on bone, or from the influence of common genetic variants on both tissues. However, the training-related changes in periosteal (but not endostial) bone volume which we describe are consistent with those in the larger study from which this sample was drawn^31^. Such changes might be more consistent with a response to muscular loading of bone, rather than gravitational. Genetic analysis would also offer further insight into the mechanisms of co-regulation.

Both muscular and gravitational loadings are likely to have been similar across individuals, and any differences irrelevant to the interpretation of results (which related to within-individual associations between muscle and bone).

A limitation in our analysis might be that we did not measure the volume of the whole RF muscle, but rather used a ten-slice sum as a proxy for this. Such an approach is valid: even single slice cross-sectional area measurements correlate very well with total RF muscle volume^43^,^44^, and such single-slice measures are of proven functional relevance^45^,^46^,^47^,^48^. We should thus emphasise that ‘whole RF volume' is not reported, and that the sum-of-slices, which we report, must, per force, represent an underestimate of that total volume. Further, it is possible that growth responses in more distal muscle regions might have differed slightly from that in the proximal 20 cm region we analysed.

In summary, we have performed the first large-scale human prospective study to investigate the relationship between regional bone architecture/BMD and muscle mass. We report, for the first time, that thigh muscle mass is lower in young healthy male smokers than in non-smokers. We found muscle volume to be related to femoral periosteal bone volume and bone mineral density. Training-related increases in muscle and bone volume were related.

## Author Contributions

Z.P. and H.M. wrote the main text, Z.P., M.K. and J.R. performed data analysis, K.E., J.P. and H.M. conceived, designed and implemented the study. All authors reviewed and approved the manuscript.

## Figures and Tables

**Table 1:** Baseline anthropomorphic, smoking, alcohol and weight bearing exercise data for the overall cohort and the muscle analysis subset. Data are mean (sd).p-values are for unpaired Student's T-test, except for # where Chi-squared test was used. Alcohol group: Low = 1–9 IU/week, Moderate = 10–21 IU/week, High > 21 iu/week. Weight-bearing exercise categorized by compound index of activity (number of sports x weekly hours of engagement): light < 19, moderate = 20–99 and heavy > 100. Data were not available in all cases for alcohol (1.5% and 1%) and weight bearing (32.3% and 33%)

	Overall cohort (n = 399)	Muscle analysis subset (n = 215)	P value
**Age**	19.9 (2.3)	20.0 (2.3)	0.659
**Height**	178.1(6.2)	178.2 (5.9)	0.842
**Weight**	73.7 (9.9)	73.8 (9.7)	0.810
**Smoking status^#^**			0.999
**Non-smokers**	278	142	
**Ex-smokers**	95	62	
**Current smokers**	26	11	
**Alcohol^#^**			0.994
**Non-drinkers**	99	43	
**Low**	129	76	
**Moderate**	132	75	
**High**	33	19	
**Weight BearingExercise^#^**			0.988
**Low**	135	68	
**Moderate**	111	65	
**High**	24	11	

**Table 2:** Comparison between total sample set (Lichfield bone study) and nested cohort. Data shown here are pre training, except when Δ is used, indicating change with training. Bone volumes are in mm^3^, and Bone Mineral Density (BMD) g/cm^2^. P values are for unpaired 2 tailed Student's t-test except for # where Wilcoxon signed rank test was used

	Baseline in overall study (n = 399)	Baseline in muscle analysis subset (n = 215)	P value	Change in overall study with training (n = 399)	Change in muscle analysis subset with training (n = 215)	P value
**Cortical Volume**	20299 ± 2505	20258 ± 2451	0.896	196 ± 81	186 ± 761	0.851
**Endosteal Volume**	6136 ± 1876	6147 ± 1839	0.973	−14 ± 662	9 ± 595	0.639
**Periosteal Volume**	26435 ± 3229	26405 ± 2988	0.902	181 ± 839	196 ± 779	0.853
**Total Hip BMD#**	1.08 ± 0.135	1.07 ± 0.13	0.490	0.019 ± 0.03	0.02 ± 0.03	0.870
**Femoral neck BMD**	0.98 ± 0.13	0.97 ± 0.13	0.561	0.01 ± 0.03	0.01 ± 0.03	0.609
**Proximal Femur BMD**	1.23 ± 0.15	1.24 ± 0.16	0.594	0.02 ± 0.04	0.02 ± 0.04	0.805
**Trochanteric BMD**	0.83 ± 0.11	0.84 ± 0.12	0.349	0.02 ± 0.02	0.02 ± 0.02	0.785
**Wards area BMD**	0.87 ± 0.14	0.89 ± 0.15	0.413	0.02 ± 0.04	0.02 ± 0.04	0.498

**Table 3:** Univariate and multivariate analysis of log_10_ [pre-training *rectus femoris* volume] and log_10_ [bone mineral density]. *denotes p < 0.05

Variable	Slope	95%CI	Intercept	R^2^	P value	Slope	Beta	P value
**Total Hip**	0.258	−0.640–0.345	−0.493	0.21	<0.001*	1.331	0.760	<0.001*
**Femoral Neck**	0.243	−0.669–0.342	−0.568	0.16	<0.001*			
**Trochanter**	0.240	−0.738–0.398	−0.567	0.14	<0.001*			
**Proximal Femur**	0.265	−0.595–0.295	−0.445	0.21	<0.001*			
**Wards Area**	0.271	−0.817	−0.609	0.13	<0.001*			

**Table 4:** Univariate analysis of log_10_ [percentage change in *Rectus femoris* volume and] and log_10_ [bone volumes]. * denotes p<0.05

Variable	Slope	95%CI	Intercept	R^2^	P value
**Dominant**					
**ΔCortical Volume**	0.105	1.474–1.564	1.519	0.278	0.001*
**ΔEndoseal Volume**	0.105	1.474–1.565	1.519	0.278	0.001*
**ΔPeriosteal Volume**	0.234	1.097–1.454	1.275	0.113	0.001*
**Non-dominant**					
**ΔCortical Volume**	0.001	1.668–1.774	1.706	0.001	0.931
**ΔEndoseal Volume**	0.029	1.724–1.776	1.705	0.062	0.001*
**ΔPeriosteal Volume**	−0.126	1.768–2.001	1.885	0.058	0.002*

## References

[b1] Doyle F., Brown J. & Lachance C. Relation between bone mass and muscle weight. The Lancet 295, 391–393, doi:http://dx.doi.org/10.1016/S0140-6736(70)91520-5 (1970). 10.1016/s0140-6736(70)91520-5 4189692

[b2] Sinaki M., McPhee M. C., Hodgson S. F., Merritt J. M. & Offord K. P. Relationship between bone mineral density of spine and strength of back extensors in healthy postmenopausal women. Mayo Clin Proc 61, 116–122 (1986). 3945109 10.1016/s0025-6196(12)65197-0

[b3] Hyakutake S., Goto S., Yamagata M. & Moriya H. Relationship between bone mineral density of the proximal femur and lumbar spine and quadriceps and hamstrings torque in healthy Japanese subjects. Calcif Tissue Int 55, 223–229 (1994). 7987737 10.1007/BF00425879

[b4] Pang M. Y. C. & Eng J. J. Muscle strength is a determinant of bone mineral content in the hemiparetic upper extremity: Implications for stroke rehabilitation. Bone 37, 103–111, doi:http://dx.doi.org/10.1016/j.bone.2005.03.009 (2005). 15869927 10.1016/j.bone.2005.03.009 PMC3167823

[b5] Nordstrom P., Thorsen K., Nordstrom G., Bergstrom E. & Lorentzon R. Bone mass, muscle strength, and different body constitutional parameters in adolescent boys with a low or moderate exercise level. Bone 17, 351–356, doi:S8756328295002405 (1995). 8573407 10.1016/s8756-3282(95)00240-5

[b6] Szulc P., Beck T. J., Marchand F. & Delmas P. D. Low Skeletal Muscle Mass Is Associated With Poor Structural Parameters of Bone and Impaired Balance in Elderly Men—The MINOS Study. Jth Bone and Miner Res 20, 721–729, 10.1359/jbmr.041230 (2005). 15824844

[b7] Forbes R. M., Cooper A. R. & Mitchell H. H. The composition of the adult human body as determined by chemical analysis. J Biol Chem 203, 359–366 (1953). 13069519

[b8] Cooper A. R., Forbes R. M. & Mitchell H. H. Further studies on the gross composition and mineral elements of the adult human body. J Biol Chem 223, 969–975 (1956). 13385244

[b9] Nordstrom P., Thorsen K., Bergstrom E. & Lorentzon R. High bone mass and altered relationships between bone mass, muscle strength, and body constitution in adolescent boys on a high level of physical activity. Bone 19, 189–195, doi:8756328296001639(1996). 8853864 10.1016/8756-3282(96)00163-9

[b10] Weeks B. K., Young C. M. & Beck B. R. Eight months of regular in-school jumping improves indices of bone strength in adolescent boys and Girls: the POWER PE study. J Bone Miner Res 23, 1002–1011, 10.1359/jbmr.080226 (2008). 18302501

[b11] Hakkinen A., Sokka T., Kotaniemi A. & Hannonen P. A randomized two-year study of the effects of dynamic strength training on muscle strength, disease activity, functional capacity, and bone mineral density in early rheumatoid arthritis. Arthritis Rheum 44, 515–522, 10.1002/1529-0131(200103)44:3 <515::aid-anr98> 3.0.co; 2–5 (2001). 11263764

[b12] Vicente-Rodriguez G., Ara I., Perez-Gomez J., Dorado C. & Calbet J. A. L. Muscular development and physical activity as major determinants of femoral bone mass acquisition during growth. Br. J Sports Med 39, 611–616, 10.1136/bjsm.2004.014431 (2005). 16118297 PMC1725300

[b13] Chen J. H., Liu C., You L. & Simmons C. A. Boning up on Wolff's Law: mechanical regulation of the cells that make and maintain bone. J Biomech 43, 108–118, 10.1016/j.jbiomech.2009.09.016S0021-9290(09)00507-7 (2010). 19818443

[b14] Guadalupe-Grau A., Fuentes T., Guerra B. & Calbet J. A. Exercise and bone mass in adults. Sports Med 39, 439–468, doi:2 (2009). 19453205 10.2165/00007256-200939060-00002

[b15] Gomez-Cabello A., Ara I., Gonzalez-Aguero A., Casajus J. A. & Vicente-Rodriguez G. Effects of training on bone mass in older adults: a systematic review. Sports Med 42, 301–325, 10.2165/11597670-000000000-00000 (2012). 22376192

[b16] Martin N. A., Zoeller R. F., Robertson R. J. & Lephart S. M. The comparative effects of sports massage, active recovery, and rest in promoting blood lactate clearance after supramaximal leg exercise. J Athel Train 33, 30–35 (1998). PMC1320372 16558481

[b17] Burr D. B. Muscle strength, bone mass, and age-related bone loss. J Bone Miner Res 12, 1547–1551, 10.1359/jbmr.1997.12.10.1547 (1997). 9333114

[b18] Rauch F. & Schoenau E. The developing bone: slave or master of its cells and molecules? Pediatr Res 50, 309–314, 10.1203/00006450-200109000-00003 (2001). 11518815

[b19] Frost H. M. Muscle, bone, and the Utah paradigm: a 1999 overview. Med Sci Sports Exerc 32, 911–917 (2000). 10795780 10.1097/00005768-200005000-00006

[b20] Rauch F., Bailey D. A., Baxter-Jones A., Mirwald R. & Faulkner R. The ‘muscle-bone unit'during the pubertal growth spurt. Bone 34, 771–775 (2004). 15121007 10.1016/j.bone.2004.01.022

[b21] Daly R. M., Saxon L., Turner C. H., Robling A. G. & Bass S. L. The relationship between muscle size and bone geometry during growth and in response to exercise. Bone 34, 281–287, 10.1016/j.bone.2003.11.009S8756328203004113 (2004). 14962806

[b22] Lang D. H. *et al.* Bone, muscle, and physical activity: structural equation modeling of relationships and genetic influence with age. J Bone Miner Res 24, 1608–1617, 10.1359/jbmr.090418 (2009). 19419307 PMC2730930

[b23] Sun X. *et al.* Genetic and environmental correlations between bone geometric parameters and body compositions. Calcif Tissue Int 79, 43–49, 10.1007/s00223-006-0041-3 (2006). 16868663

[b24] Karasik D. & Kiel D. P. Genetics of the musculoskeletal system: a pleiotropic approach. J Bone Miner Res 23, 788–802, 10.1359/jbmr.080218 (2008). 18269309 PMC3280426

[b25] Deng F. Y. *et al.* Bivariate whole genome linkage analysis for femoral neck geometric parameters and total body lean mass. J Bone Miner Res 22, 808–816, 10.1359/jbmr.070303 (2007). 17352645

[b26] Garland T. Jr *et al.* Evolution of a small-muscle polymorphism in lines of house mice selected for high activity levels. Evolution 56, 1267–1275 (2002). 12144025 10.1111/j.0014-3820.2002.tb01437.x

[b27] Eleftheriou K. I. *et al.* Bone structure and geometry in young men: the influence of smoking, alcohol intake and physical activity. Bone 52, 17–26, doi:S8756-3282(12)01227-610.1016/j.bone.2012.09.003 (2013). 22985892 10.1016/j.bone.2012.09.003

[b28] Saito T. *et al.* Relationship between cigarette smoking and muscle strength in Japanese men. J Prev Med Public Health 45, 381–386, 10.3961/jpmph.2012.45.6.381 (2012). 23230468 PMC3514468

[b29] Kok M. O., Hoekstra T. & Twisk J. W. The longitudinal relation between smoking and muscle strength in healthy adults. Eur Addict Res 18, 70–75, doi:00033360010.1159/000333600 (2012). 22178906 10.1159/000333600

[b30] Ferretti J. L., Cointry G. R., Capozza R. F. & Frost H. M. Bone mass, bone strength, muscle-bone interactions, osteopenias and osteoporoses. Mech Ageing Dev 124, 269–279, doi:S004763740200194X (2003). 12663124 10.1016/s0047-6374(02)00194-x

[b31] Eleftheriou K. I. *et al.* The Lichfield bone study: the skeletal response to exercise in healthy young men. J Appl Physiol 112, 615–626, 10.1152/japplphysiol.00788.2011 (2012). 22114178 PMC3289434

[b32] Rawal J. *et al.* Relationship between calcaneal quantitative ultrasound and hip dual energy X-ray absorptiometry in young healthy men. Osteoporos Int 23, 1947–1956, 10.1007/s00198-011-1853-1 (2012). 22222754

[b33] Williams A. G., Rayson M. P. & Jones D. A. Effects of basic training on material handling ability and physical fitness of British Army recruits. Ergonomics 42, 1114–1124, 10.1080/001401399185171 (1999). 10504891

[b34] Williams A. G., Rayson M. P. & Jones D. A. Resistance training and the enhancement of the gains in material-handling ability and physical fitness of British Army recruits during basic training. Ergonomics 45, 267–279, 10.1080/00140130210123525 (2002). 12028724

[b35] Leite M. L. & Nicolosi A. Lifestyle correlates of anthropometric estimates of body adiposity in an Italian middle-aged and elderly population: a covariance analysis. Int J Obes (Lond) 30, 926–934, 10.1038/sj.ijo.0803239 (2006). 16432539

[b36] Debigare R., Cote C. H. & Maltais F. Peripheral muscle wasting in chronic obstructive pulmonary disease. Clinical relevance and mechanisms. Am J Respir Crit Care Med 164, 1712–1717 (2001). 11719314 10.1164/ajrccm.164.9.2104035

[b37] Gosselink R., Troosters T. & Decramer M. Peripheral muscle weakness contributes to exercise limitation in COPD. Am J Respir Crit Care Med 153, 976–980 (1996). 8630582 10.1164/ajrccm.153.3.8630582

[b38] Orozco-Levi M. *et al.* Injury of peripheral muscles in smokers with chronic obstructive pulmonary disease. Ultrastruct Path 36, 228–238, 10.3109/01913123.2012.668611 (2012). 22849524

[b39] Castillo E. M. *et al.* Sarcopenia in elderly men and women: the Rancho Bernardo study. Am J Prev Med 25, 226–231 (2003). 14507529 10.1016/s0749-3797(03)00197-1

[b40] Petersen A. M. *et al.* Smoking impairs muscle protein synthesis and increases the expression of myostatin and MAFbx in muscle. Am J Physiol Endocrinol Metab 293, E843–848, doi:00301.2007 10.1152/ajpendo.00301.2007 (2007). 17609255 10.1152/ajpendo.00301.2007

[b41] Frost H. M. & Schonau E. The "muscle-bone unit" in children and adolescents: a 2000 overview. J Pediatr Endocrinol Metab: JPEM 13, 571–590 (2000). 10.1515/jpem.2000.13.6.571 10905381

[b42] Gillespie J. A. The influence of sex hormones on the bony changes occurring in paralysed limbs. J Endocrinol 11, 66–70 (1954). 13184082 10.1677/joe.0.0110066

[b43] Walton J. M., Roberts N. & Whitehouse G. H. Measurement of the quadriceps femoris muscle using magnetic resonance and ultrasound imaging. Br. J Sports Med 31, 59–64 (1997). 9132215 10.1136/bjsm.31.1.59 PMC1332478

[b44] Mathur S., Takai K. P., Macintyre D. L. & Reid D. Estimation of thigh muscle mass with magnetic resonance imaging in older adults and people with chronic obstructive pulmonary disease. Phys Ther 88, 219–230 (2008). 18056754 10.2522/ptj.20070052

[b45] Seymour J. M. *et al.* Ultrasound Measurement of Rectus Femoris Cross-Sectional Area and the Relationship to Quadriceps Strength in Chronic Obstructive Pulmonary Disease. Thorax 64, 418 (2009). 19158125 10.1136/thx.2008.103986

[b46] Shrikrishna D. *et al.* Quadriceps wasting and physical inactivity in patients with COPD. Eur Respir J, 10.1183/09031936.00170111 (2012). 22362854

[b47] Sarianna S. & Harri S. Ultrasound imaging of the quadriceps muscle in elderly athletes and untrained men. Muscle Nerve 14, 527–533 (1991). 1852160 10.1002/mus.880140607

[b48] Puthucheary Z. A. *et al.* Acute skeletal muscle wasting in critical illness. JAMA 310, 1591–1600, 10.1001/jama.2013.278481 (2013). 24108501

